# The role of the renin–angiotensin system blocking in the management of atrial fibrillation

**DOI:** 10.3109/21556660.2012.672353

**Published:** 2012-03-05

**Authors:** Brett Cliff, Naveed Younis, Salam Hama, Handrean Soran

**Affiliations:** 1 University Department of Medicine, Central Manchester University Hospitals NHS Foundation Trust, ManchesterUK; 2 Department of Diabetes and Endocrinology, South Manchester University Hospitals NHS Foundation Trust, ManchesterUK; 3 Cardiovascular Research Group, School of Biomedicine, Core Technology Facility (3rd Floor), University of Manchester, ManchesterUK; 4 University Department of Medicine, Central Manchester University Hospitals, ManchesterUK

**Keywords:** Angiotensin converting enzyme inhibitors, Angiotensin II receptor blockers, Atrial fibrillation, Renin–angiotensin system

## Abstract

**Objective:**

To review current available evidence for the role of renin–angiotensin system blockade in the management of atrial fibrillation.

**Method:**

We conducted a PubMed and Medline literature search (January 1980 through July 2011) to identify all clinical trials published in English concerning the use of angiotensin converting enzyme inhibitors or angiotensin II receptor blockers for primary and secondary prevention of atrial fibrillation. We also discussed renin–angiotensin system and its effects on cellular electrophysiology.

**Conclusion:**

The evidence from the current studies discussed does not provide a firm definitive indication for the use of angiotensin converting enzyme inhibitors or angiotensin II receptor blockers in the primary or secondary prevention of atrial fibrillation. Nevertheless, modest benefits were observed in patients with left ventricular dysfunction. In view of the possible benefits and the low incidence of side-effects with angiotensin converting enzyme inhibitors and angiotensin II receptor blockers, they can be given to patients with recurrent AF, specifically those with hypertension, heart failure and diabetes mellitus.

## Introduction

Atrial fibrillation (AF) is the most common sustained cardiac arrhythmia^1^. Prevalence of AF increases with age^2–4^. Individuals with AF have a two-fold increased mortality and five-fold increased stroke risk^3,5^. Some authors have indicated that AF could be causing those with a history of hypertension to have an even more elevated BP^6^. Causes of AF are summarized in Table 1^1–5,7^.

**Table 1.** Causes of atrial fibrillation.

Cardiac	Non-cardiac
Valvular disorders	Thyrotoxicosis
Left ventricular hypertrophy	Pulmonary embolism
Heart failure	Rare mutations in a series of ion channels
Coronary artery disease	Sepsis
Congenital heart disease	Pneumonia
Sick sinus syndrome	
Pericarditis	
Myocarditis	
Hypertension	

AF is generally thought to be caused by multiple re-entrant wavelets that propagate randomly throughout the atria^8^. Atrial tachycardias, which include atrial fibrillation, can cause electrical remodeling, that produce multi-circuit re-entry AF^9^. This remodeling also acts on a cellular level resulting in decreased contractility and leading to atrial cardiomyopathy. Many other theories have been hypothesised for mechanisms contributing to development of AF. It has been suggested that high angiotensin II levels can cause atrial fibrosis, which may play a role in AF^10^. Some studies suggest that paroxysmal AF is precipitated by premature beats^11–13^. Others propose some input from high vagal tone and a degree of subclinical myocarditis^14,15^. It is clear that electrical and mechanical remodeling occurs following arrhythmia that can lead to sustained AF.

The renin–angiotensin system (RAS) has been shown to play an important role in the onset of AF. RAS activation cause the electrical and structural remodeling of diseased atria by altering the balance between atrial currents, decreasing the cardiac action potential duration^16,17^ and causing fibrosis via disturbing the continuous cable-like arrangement of cardiomyocytes^18^. Additionally, RAS activation may cause structural remodeling due to mitogen-activated protein kinase (MAPK) expression and reduction of collagenase activity^19^. This can lead to fibrosis of the atrial myocardia^20^ and congestive heart failure (CHF)^21^.

Thus lots of interest as to whether blocking RAS activity, with angiotensin converting enzyme inhibitors (ACE-I) or angiotensin II receptor blockers (ARBs), could be used to prevent electrical and structural remodeling has been generated.

## The roles of renin–angiotensin system

**Figure 1.**
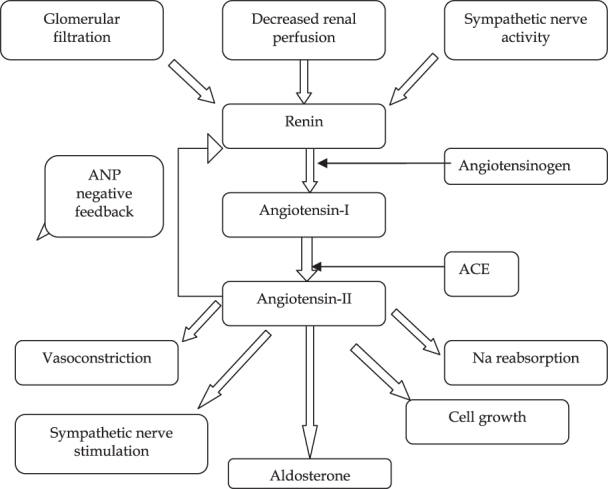
Renin–angiotensin system.

The RAS plays a vital physiological role. It promotes vascular growth and regeneration, salt retention and vasoconstriction^22^. Renin, a proteolytic enzyme, is secreted from the juxtaglomerular apparatus in the kidney in response to a decrease in blood pressure detected by baroreceptors in the vessels, activation of the sympathetic nervous system and when the macular densa responds to a decrease in the amount of NaCl in nephrons. Subsequently, renin is converted to angiotensin I by angiotensinogen, then broken down to angiotensin II by angiotensin converting enzyme (ACE). This then acts on angiotensin (AT1) and AT2 receptors^23^ (Figure 1). All classic physiological effects of angiotensin II such as sodium and water retention, sympathetic activation, vasoconstriction, aldosterone and vasopressin release are mediated by the AT1 receptor. Via its AT1 receptor, angiotensin II is also involved in cell proliferation, left ventricular (LV) hypertrophy, nephrosclerosis, vascular media hypertrophy, endothelial dysfunction, neointima formation and processes leading to atherothrombosis, as well as in the modulation of various biological processes involved in development, cell differentiation, tissue repair and apoptosis^23,24^. Angiotensin II is also a powerful dipsogen^25^ which acts on the subfornical organ in the brain to increase blood volume by stimulating the perception of thirst^26^. The posterior pituitary gland is stimulated by the hypothalamus to secrete antidiuretic hormone (vasopressin). This acts on V2 receptors in the basolateral membrane of the distal tubules and collecting ducts in a nephron, resulting in an overall reduction in urinary output^27^. Furthermore, Weisinger and Cooper have suggested that body fat and obesity are also associated with increased levels of ACE and angiotensinogen, leading to an elevation in the blood pressure^28^. The actions of the RAS are summarized in Figure 1.

Aldosterone is secreted form the adrenal cortex and acts on a number of target organs, including the kidney and the transporting epithelia of bladder and colon. Aldosterone enhances sodium re-absorption from the collecting tubules, thus leading to increased blood pressure^29^. Blockade of the aldosterone secretion has shown to restore the baroreceptor reflex, which tends to improve the variation in heart rate in heart failure patients^30^. Increased production of aldosterone is associated with hypertension and cardiac failure^31^. In fact a recent study has shown that even mild plasma elevations of aldosterone concentration is associated with a higher risk of fatal cardiovascular disease^32^. Spironolactone, a competitive aldosterone inhibitor, has shown benefits in the treatment of cardiovascular disease^33^. It blocks aldosterone’s mineralocorticoid effects on the renal tubule. Eplerenone, a selective aldosterone blocker, is also proven to have cardiovascular benefits^34^. Different effects of aldosterone are summarized in Table 2.

**Table 2.** Actions of aldosterone.

(1) Regulation of K^+^ homeostasis
(2) NaCl retention which helps to prevent arterial hypotension^35^ by retaining blood volume
(3) Non-geonomic actions which recent evidence suggests maybe attributable to the classical mineralocorticoid receptors^36^
(4) Improvement of endothelial cell dysfunction and a reduction in vascular smooth muscle cells and myocyte remodeling. This contributes to improved vascular compliance, which in turn reduces the development of left ventricular dysfunction and end-organ damage^30^

Traditionally there are two ways of managing AF, rate or rhythm control. A recent study suggested that in a patient with AF after myocardial infarction (MI), there is nearly a two-fold excess in early mortality (0–45 days post-MI) with anti-arrhythmic drug-based rhythm control, as opposed to rate-control medication^37^. The AFFIRM trial^38^ compared rate verses rhythm control for the treatment of AF. The results showed no survival advantage with either strategies, but potential drawbacks such as adverse drug effects and increased rate of hospitalization (80.1 vs. 73.0%, *p* < 0.001) in the rhythm group.

Interestingly, acute angiotensin II infusion has been shown to increase intra-atrial pressure and shorten both the atrial action potential duration and refractory period^39^, though this has not been the case in all studies^40^. In addition to influencing electrical remodeling, angiotensin II may augment the structural remodeling of AF through the endorsement of apoptosis and fibrosis^41,42^. Likewise, aldosterone may contribute to structural remodeling associated with AF by enhancing atrial fibrosis^43^. Thus, theoretically, RAS inhibition should help to prevent atrial remodeling and AF development.

## Effects RAS on cellular electrophysiology

Ion channels modulations secondary to dilatation of atria may play an important role in AF and its maintenance^44,45^. Angiotensin II stimulation increase T-type calcium channel *(I*_Ca,T_)^46^ and L-type calcium channel (*I*_Ca,L_) through protein kinase C (PKC) dependent pathways^47^ and *I*_Ca,T_ blockade is reported to prevent AF-substrate development^48^. Therefore, it is tempting to conclude that inhibition RAS might be beneficial by preventing angiotensin II-mediated *I*_Ca,T_ increases. Mibefradil, a drug with strong *I*_Ca,T_ blocking properties, prevents atrial tachycardia induced AF promoting electrophysiological remodelling^49^ but no similar benefits observed with ACE-I^50^.

Transcriptional transient outward (*I*_to_) down regulation is a hallmark of AF-induced electrical remodelling^51^. The AT_1_-receptor regulates *I*_to_ cell-surface expression^52^ and also contributes to intracellular *I*_to_ reduction. Furthermore, angiotensin II increased the rapid delayed-rectifier current (*I*_Kr_) in guinea pig ventricular myocytes, while the slow component (*I*_Ks_) is decreased^53^.

*In vitro* studies showed different effects of ARBs. Losartan blocks currents carried by HERG-related gene corresponding to *I*_Kr_, KvLQT1, *I*_Ks_ and hKv1.5 subunits^54^. Candesartan, eprosartan, and irbesartan inhibit currents carried by hKv1.5, KvLQT1 + mink, and rKv4.3 subunits^55^. Whereas, irbesartan blocks rKv4.3 and hKv1.5 at therapeutic concentrations, blockade of HERG and KvLQT1 + minK currents required supra-therapeutic concentrations^56,57^. ARBs may be beneficial in acute atrial remodeling by directly inhibiting repolarizing K^+^ currents. Some ARBs inhibit Kv1.5 currents, which stimulate the atrially expressed sustained outward potassium current (Kur)^58,59^, an emerging target for AF therapy^59^. Overall, these observations imply that the renin–angiotensin system could play an important role if the cellular electrophysiology of AF via ion-channel modulation, impulse propagation and facilitate re-entry and inhibiting RAS may have possible advantages.

## Primary prevention of AF using ACE-I/ARB

### Left ventricular dysfunction

The randomized TRACE trial^60^ used trandolapril in patient with LV dysfunction and sinus rhythm following an acute MI. The results showed a significant reduction (2.8 vs. 5.3%) in the incidence of AF in the 2–4 years follow-up period in the trandolapril group. There was a similar picture in the retrospective analysis of the SOLVD randomized trial^61^ and Val-HeFT^62^, both studies recruited patient with chronic LV dysfunction. The SOLVD trial showed that only 5.4% of those treated with enalapril developed AF, by the mean follow-up point of 2.9 years, compared to 24% developing new onset AF in the placebo group.

The CHARM study also supported the above trials conclusion. It enlisted over 7000 patients, the majority of which did not have AF at baseline. Patient with both diastolic and systolic dysfunction were recruited. At an average follow-up period of 37.7 months, an episode of AF had been reported in 5.55% in the candesartan group compared to 6.74% in the placebo group (*p* = 0.048)^63^.

A meta-analysis of 11 trials by Healey in 2005^64^, summarized that both ARB and ACE-I are effective in the primary prevention of AF with a relative risk reduction [RRR] of 0.28 (95% CI 0.15–0.40), but only in patients with systolic LV dysfunction or hypertrophy. These clinical trials are summarized in Table 3.

**Table 3.** Summary of clinical trials.

First author/study, year published (reference number)	Population	Number of patients	Drug used	Control drug	Mean follow-up	Finding	*p*-value
**Left ventricular dysfunction**
Pedersen (TRACE), 1999	LVD secondary to MI	1577	Trandolapril	Placebo	2–4 years	Reduced new onset AF 5.3 vs. 2.8% RR 0.55; 95% CI 0.33–0.91	<0.05
Vermes (SOLVD), 2003	LVD, CHF	374	Enalapril	Placebo	3.3 years	Reduced new onset AF 24 vs. 5.45% RR 0.30; 95% CI 0.15–0.58	<0.0001
Maggioni (Val-HeFT), 2005	LVD	4395	Valsartan	Placebo	23 months	Reduced new onset AF 7.95 vs. 5.12% RR 0.70; 95% CI 0.57–0.87	<0.0002
Ducharme (CHARM), 2006	LVD, CHF, PLVF	6379	Candesartan	Placebo	37.7 months	Reduced new onset AF 6.15 vs. 5.55% RR 0.822; 95% CI 0.662–0.998	0.048
**Hypertension** L'Allier, 2004	HTN, retrospective study of a cohort of 10,926 patients	10926	ACE inhibitors	CCB	4.5 years	Reduced new onset AF 22.1 vs. 19.4% RR 0.95; 95% CI 0.74–0.97	0.018
Wachtell (LIFE), 2005	HTN, LVH	8815	Losartan	Atenolol	4.9 years	Reduced new onset AF 10.1 vs. 6.8% RR 0.67; 95% CI 0.56–0.84	<0.001
Hansson (CAPP), 1999	HTN	10,985	Captopril	β-blockers and/or diuretics	6.1 years	No difference in new onset AF RR 0.87%; 95% CI 0.68–1.11	NS
Hansson (STOP-H2), 1999	HTN	6614	Enalapril or CCBs	β-blockers and/or diuretics	5.0 years	No difference in new onset AF between the three groups RR 1.10; 95% CI 0.93–130	NS
Julius (VALUE), 2004	HTN	15313	Valsartan	Amlodipine	4.2 years	No difference in new onset AF RR 1.19; 95% CI 0.96–1.47	NS
Heckbert, 2009	HTN	2322	Varied in study	Nil	NS	Reduced episodes of new onset AF. adjusted OR 0.63, 95% CI 0.44–0.91	NS
Yamashita T, (J-RHYTHM II), 2010	HTN	318	Candesatran	Amlodipine	1 year	No difference in new onset AF 3.8 ± 5.0 in the ARB group vs. 4.8 ± 6.3 in the CCB group	NS
ONTARGET investigators, 2008	HTN	25620	Ramipril or temlisartan		56 months	No difference in new onset AF 16.5 vs. 16.7%; RR 1.01; 95% CI 0.94–1.09	NS
Yusuf (Transcend), 2008	HTN, intolerant to ACE-i	5926	Temlisartan	Placebo	56 months	No difference in primary events. 15.7 vs. 17.0%, HR 0.92, 95% CI 0.81–1.05	0.216
**Post-MI and CABG**
Pizetti (GISSI), 2001 (25)	Post-MI	17,749	Lisinopril or lisinopril and nitrates	Nitrates	42 days	No significant reduction in new onset AF RR 0.93; 95% CI 0.84–1.03	NS
Mathew 2004 (26)	Post-CABG	4657	ACE inhibitors	No ACE inhibitors	NS	Postoperative withdrawal of ACE inhibitor increased risk of AF OR 1.69; 95% CI 1.38–2.08 Reduced risk of AF with pre and Postoperative administration of ACE Inhibitors OR 0.62; 95% CI 0.48–0.79	0.001 <0.001
Mathew	Undergoing CABG	4657 (626 given ACE-I)	ACE-I	–	NS	Reduced incidence of new onset AF. CI (0.62 (0.48–0.79)	<0.001
**Recurrence of AF**
Van den Burg, 1995	Post electrical CV, CHF	30	Lisinopril	Placebo	84 days	Sinus rhythm maintained in 71% in the lisinopril vs. 36% in the placebo group	NS
Madrid, 2002	AF, post pharmacological and electrical CV	154	Irbesartan and amiodarone	Amiodarone	254 days	More patients in the Irbesartan group remained in SR 84.79 vs. 63.16%. This remained statistically significant on multivariate analysis RR 0.19; 95% CI 0.04–0.86	0.031
Ueng, 2003	Post electrical CV	125	Enalapril and amiodarone	Amiodarone	270 days	More patients in the enalapril group remained in SR 74.3 vs. 57.3% HR 0.60; 95% CI 0.25–0.91*	0.021
Yin, 2006	Paroxysmal AF	177	Losartan and amiodarone, perindopril and amiodarone	Amiodarone	2 years	Significant reduction in recurrence of AF in losartan and amiodarone (19%) (RR 0.46; 95% CI 0.17–0.75) and perindopril and amiodarone (24%) groups (RR 0.58; 95% CI 0.20–0.78) vs. 41% in the amiodarone	0.006 0.008
Murray, 2004	AF	732	ACE inhibitors or ARBs	No ACE inhibitors or ARBs	3.5 years	No difference in AF recurrence HR 0.91; 95% CI 0.77–1.09	NS
Van Noord, 2005	AF	107	ACE inhibitors treatment before start of the present episode of AF	No ACE inhibitors	1 month	Pre-treatment with ACE inhibitors improved acute success of ECV. Recurrence at 1 month was similar RR 0.98; 95% CI 0.75–1.66	0.04 NS
Richter, 2006	AF	234	Statins, ACE inhibitors or ARBs. Statins plus an ACE inhibitor or ARBs.	NA	12.7 months	No difference in AF recurrence after ablation	NS
Aenn, 2004	AF	196	ACE inhibitors/ARBs	NA	2.2 years	ACE inhibitors/ARBs pre-treatment reduced risk of post ablation AF RR 0.55; 95% CI 0.31–0.97	0.04
CAPRAF	AF prior to ECV	124	Candesartan	Placebo	29 days	No difference in recurrence of AF	
Fogari, 2008	HTN and DM and AF	296	Valsartan	Atenolol	1 year	Fewer recurrences of AF 20.3 vs. 34.1%	<0.01
Fogari, 2008	HTN	369	Valsartan or ramipril	Amlopine	1 year	Fewer recurrences in AF with trial drugs. 16.1 vs. 27.9 vs. 47.4%	<0.01

ACEI, angiotensin-converting enzyme inhibitors; ARBs, angiotensin receptor blockers; AF, atrial fibrillation; CABG, coronary artery bypass graft; CHARM, Candesartan in Heart failure: Assessment of Reduction in Mortality and morbidity program; CAPP, Captopril Prevention Project; CCBs, calcium channel blockers; CHF, congestive heart failure; EC, electrical cardioversion; GISSI, The Gruppo Italiano per lo Studio della Sopravvivenza nell'Infarto Miocardio trial; HTN, hypertension; LVD, left ventricular dysfunction; LIFE, Losartan Intervention for End Point Reduction in Hypertension trial; LVEF, left ventricular ejection fraction; LVH, left ventricular hypertrophy; n/a, not available; NS, not significant; PLVF, preserved left ventricular function; MI, myocardial infarction; SOLVD, Study of Left Ventricular Dysfunction trial; STOP-H2, Swedish Trial in Old Patients with Hypertension-2 study; TRACE, Trandolapril Cardiac Evaluation Study; Val-HeFT, Valsartan Heart Failure Trial; VALUE, Valsartan Antihypertensive Long-term Use Evaluation. *Subgroup of patients with left atrial size >40 mm

### Hypertension

L'Allier *et al*.^65^ performed a retrospective study on a medical and pharmacy claims database. They compared the incidence of AF in 10,926 hypertensive patients who were prescribed an ACE-I vs. a calcium channel blocker (CCB). At an average follow-up of 4.5 years, there was a significantly reduced incidence of AF in the ACE-I group (17.9 per 1000 patient-years) than the CCB group (18.9 per 1000 patient-years). The hazard ratio for the patient treated with ACE-I was 0.85 (95% CI 0.74–0.97). These results were supported by a post hoc analysis from the LIFE trial^66,67^. Hypertensive patients with electrocardiograph (ECG) evidence of LV hypertrophy and a mean BP of 177/97 mmHg were randomly given losartan or atenolol. New onset AF occurred in 150 patients randomized to losartan and 221 patients in the atenolol arm. Relative risk reduction using losartan was 0.67 (95% CI 0.55–0.83, *p* < 0.001).

Heckbert and colleagues^68^ observed hypertensive patients without heart failure to determine if treatment with ACE-I and/or ARB’s, as opposed to diuretics, is associated with fewer occurrences of AF. The results showed that single users of ACE-I/ARB produced fewer episodes than single users of a diuretic (adjusted OR 0.63, 95% CI 0.44–0.91).

In contrast, the CAPPP trial^69^ randomized 10985 hypertensive patients to either a Captopril or a beta blocker and diuretic group. The results showed no difference in new onset AF between the two groups. The VALUE trial^70^ randomized 15,245 patients with hypertension to received either valsartan or amlodipine, there were no difference in the occurrence of AF. The STOP Hypertension-2^71^ studied new onset AF in a population of 6614 hypertensive patients. The patients were randomly assigned to CCB, ACE-I or beta blocker and/or diuretics. The study found no difference in new onset AF between all 3 groups. The J-RHYTHM II^72^ studied 318 hypertensive patients who were randomly assigned candesartan or amlodipine. There was no significant difference in AF frequency between the two. Amlodipine had a greater effect on lowering the blood pressure.

Over all, the hypertensive trials gave inconsistent results. Possible reasons for this includes the fact that none of the final 3 trials were placebo controlled and patients in CAPP and STOP Hypertension-2 had less severe hemodynamic abnormalities as determined from echocardiography parameters. In the VALUE study there were significantly higher initial blood pressures in the valsartan group. The CAPPP trial administered captopril as a once or twice daily dose, however, it is now well recognized that captopril has a short duration of action, implying the possibility of an inadequate dose.

In the Study on Cognition and Prognosis in the Elderly (SCOPE), elderly hypertensive patients were investigated to determine the effect of candesartan on reducing the risk of cerebrovascular events. It demonstrated a significant reduction in nonfatal strokes in those treated with an candesartan compared with a placebo^73^. A possible theory is reduction in AF in these patients could have contributed to reducing nonfatal strokes, however there is no evidence to support this.

### Post-myocardial infarction and coronary artery bypass graft

There have been two studies that observed the risk of AF following an MI in patients on ACE-I. The GISSI-3 study^74^ looked at the effects of lisinopril and/or transdermal glycerol nitrate on mortality and LV function after acute MI. There was no significant reduction in AF with 6 weeks of treatment. The TRACE study^60^ looked at patients given trandolapril 3–7 days following MI. Unlike the previous trial, TRACE study showed a 48% relative risk reduction in incidence of AF. The TRACE study enrolled only patients with LV dysfunction, whereas the GISSI-3 had mainly patients with no evidence of heart failure. This, as shown in the previous section on LV hypertrophy, may hold an important key to the difference in results.

A further multicenter study analyzed 4657 patients who were undergoing coronary artery bypass graft (CABG). Those treated with ACE-I had significantly less postoperative AF occurrences and furthermore in those who had post-operative withdrawal of ACE-I had increased AF occurrence^75^.

## Secondary Prevention

Madrid *et al*.^76^ analyzed 154 patients who had been electrically cardioverted for persistent AF, were then randomly prescribed amiodarone alone or in combination with irbesartan. The results showed those additionally treated with irbesartan had a significantly higher probability of remaining in sinus rhythm at 2 months (84.75% compared to 63.17%, *p* = 0.008), and at a median follow-up of 254 days (79.52% to 55.91%, *p* = 0.007).

A Similar trial by Ueng *et al*.^77^ randomized patients to amiodarone with or without enalapril 4 weeks prior to electric cardioversion. Those treated with the ACE-I had a lower rate of immediate recurrence (4.3% vs. 14.7%, *p* = 0.067) and a higher probability of remaining in sinus rhythm at a median follow-up of 270 days (74.3% vs. 5703%, *p* = 0.021).

A prospective randomized trial^78^ compared the incidence of AF in 177 patient with lone paroxysmal AF. Patients were randomized to amiodarone alone, amiodarone plus losartan or amiodarone plus perindopril. The data showed a significant reduction in AF recurrence in both the losartan (*p* = 0.006) and perindopril (*p* = 0.04) groups compared to the amiodarone alone group. At the 2 year follow-up, the left atrial size in those randomized to losartan or perindopril was significantly smaller. In summary, these results demonstrate a significant reduction in AF recurrence when amiodarone is combined with an ACE-I or ARB, rather than given alone.

Van Noord *et al*.^79^, studied 107 consecutive patients undergoing electrical cardioversion. Those who had ACE-I started before cardioversion showed significantly greater acute success of the treatment (95% CI 1.3–26.1, *p* > 0.05), although it did not help to reduce recurrence. In contrast another study^80^, suggested that the use of ACE-I/ARBs or diuretics before ablation resulted in a significantly lower rate of AF recurrence post treatment (*p* = 0.04; RR 0.55; 95% CI 0.31–0.97 and *p* < 0.01; RR 0.28; 95% CI 0.13–0.63, respectively). However, a retrospective analysis by Richter *et al.*^81^ showed conflicting results. They looked at recurrence following AF ablation and discovered no improvement to recurrence rates with routine ACE-I, ARB and statin use.

The CAPRAF trial^82^ randomized 124 patients with AF, prior to electrical cardioversion, to receive placebo or candesartan. Candesartan was associated with improved cardioversion outcome, nonetheless it decreased the fibrillatory rate, but this effect was restricted to patients with high baseline fibrillatory rates. Fogari^83^ studied the recurrence rate of AF in hypertensive patients with well-controlled type II diabetes mellitus. One arm received valsartan and amlodipine while the other was treated with atenolol and amlodipine. Both groups showed a statistically significant decrease in blood pressure with no difference between the two groups. Over a year’s follow-up, the valsartan/amlodipine combined group showed a statistically lower recurrence rate (20.3%) than the atenolol and amlodipine combination (34.1%). The group also investigated the recurrence of AF in 369 patients with mild hypertension. All patients were in sinus rhythm at but had at least two recorded episodes of AF in the preceding 6 months^84^. They were randomized to receive a year of amlodipine, valsartan or ramipril. All three groups showed a significant reduction in the BP (*p* < 0.001). There was a significant decrease in AF recurrence in the valsartan (16.1%) and ramipril (27.9%) groups when compared to amlodipine (47.4%). Fogari’s two studies^83,84^ support the theory that an ARB or an ACE-I leads to a lower rate of recurrence. It also suggests that an ARB maybe more effective.

A retrospective analysis of the AFFIRM study demonstrated no difference in recurrence rates of AF between the 421 patients treated with ARB/ACE-I^38^, and the 732 patients without. The study, however, showed that those with congestive heart failure or impaired LV dysfunction did have a lower risk of recurrence of AF if treated with either an ARB or ACE-I.

The GISSI-AF^85^ study looked at 1442 patients with recurrent AF. They were randomized to a placebo or valsartan group. At 1 year, there was no recurrence difference in the two groups. There was a slight but non-significant beneficial effect in those with LV dysfunction or HF. A possible reason for the lack of effect in the valsartan group could be due to optimal background therapy.

The active-I trial^86^ looked at a group of 9016 subjects with AF who were randomized to placebo or irbesartan. The patients receiving irbesartan showed a significant decrease in hospitalization from HF (HR 0.86, 95% CI 0.76–0.98, *p* = 0.018), and also a 40% reduction in stroke.

## Discussion

The evidence from the current studies discussed does not provide a firm definitive indication for the use of ARB/ACE-I to prevent new-onset AF, recurrence of AF or treat established AF. Inconsistent findings between different studies are likely to be related to variation in the number of individuals in each trial and trial methodology. Modest benefits in terms of reduction in AF onset were seen in those with LV dysfunction^60–63^. A couple of meta-analyses estimated the magnitude of RAS blocking on AF and aimed at identifying patient subgroups most likely to benefit^64,84^ revealed that patients with LV dysfunction or LVH tend to benefit the most.

## Conclusion

In view of the possible benefits and general low incidence of side-effects, ACE-I and ARB can be used in patients with recurrent AF and those at risk of AF, specifically those with hypertension, heart failure and diabetes mellitus. Future randomized control studies should aim to clarify the benefits from the use of ARB/ACE-I and determine a definitive indication for their use.
